# The Role of SGLT2 Inhibitors in Heart Failure: A Systematic Review and Meta-Analysis

**DOI:** 10.1155/2021/9927533

**Published:** 2021-08-19

**Authors:** Vasiliki Tsampasian, Ranu Baral, Rahul Chattopadhyay, Maciej Debski, Shruti S Joshi, Johannes Reinhold, Marc R Dweck, Pankaj Garg, Vassilios S Vassiliou

**Affiliations:** ^1^Department of Cardiology, Norfolk and Norwich University Hospitals, Norwich, UK; ^2^Department of Cardiology, Cambridge University Hospitals, Cambridge, UK; ^3^Norwich Medical School, University of East Anglia, Norwich, UK; ^4^University of Edinburgh/British Heart Foundation Centre for Cardiovascular Science, Edinburgh, UK

## Abstract

**Aims:**

Recent randomised controlled trials (RCTs) have shown a significant prognostic benefit of sodium-glucose cotransporter 2 (SGLT2) inhibitors in the cardiovascular (CV) profile of patients with diabetes. This systematic review and meta-analysis aim to provide a concise evaluation of all the available evidence for the use of these agents in patients with heart failure (HF) regardless of their baseline diabetes status.

**Methods and Results:**

PubMed, Web of Science, and Cochrane library databases were systematically searched from inception until November 20^th^ 2020. Eight studies consisting of 13,275 patients were included in the meta-analysis. For the total population, SGLT2 inhibitors reduced the risk of all-cause mortality (HR: 0.83; 95% CI: 0.75–0.91; *I*^2^ 0%), hospitalisation for HF (HR: 0.68; 95% CI: 0.61–0.75; *I*^2^: 0%), CV death (HR: 0.82; 95% CI: 0.74–0.92; *I*^2^: 0%), and hospitalisation for HF or CV death (HR: 0.72; 95% CI: 0.66–0.78; *I*^2^: 0%). Subgroup analyses of the total population according to the diabetes status showed that SGLT2 inhibitors significantly reduced the risk of hospitalisation for HF (HR: 0.68; 95% CI: 0.61, 0.75; *I*^2^: 0%), as well as the risk of hospitalisation for HF or CV death (HR: 0.72; 95% CI: 0.66, 078; *I*^2^: 0%) and CV death (HR: 0.82; 95% CI: 0.74, 0.91; *I*^2^: 0%).

**Conclusions:**

The results of this meta-analysis confirm the growing evidence in the literature of the favourable profile of SGLT2 inhibitors in cardiovascular outcomes and mortality in patients with heart failure regardless of the baseline diabetes status. This systematic review has been registered with PROSPERO (CRD42021224777).

## 1. Introduction

Over the recent years, large randomised controlled trials have demonstrated that sodium-glucose cotransporter 2 (SGLT2) inhibitors improve cardiovascular outcomes irrespective of diabetes, including risk of hospitalisation for heart failure (HHF), cardiovascular death, and all-cause mortality [1–4]. Being a glucose-lowering medication, SGLT2 inhibitors proved to have a significant role in reducing major adverse cardiovascular outcomes and hospitalisation for heart failure initially in patients with diabetes. The magnitude of their impact has been subsequently shown to be potentially independent—or, at least, separated—from their glucose-lowering value with a few hypotheses behind the exact mechanisms of their actions [5, 6]. The rapid accumulation of such evidence showing their favourable impact has triggered further research exploring their potential on cardiovascular outcomes and mortality in larger cohorts, not necessarily limited to diabetic populations. In response to this, two large randomised controlled studies investigated the impact of SGLT2 inhibitors in heart failure patients, with the cohort being comprised of patients with and without diabetes [7, 8].

With an increasing number of trials reporting on SGLT2 inhibitors in patients with and without diabetes, the goal of this systematic review and meta-analysis is to provide a concise evaluation of all the available evidence so far and analyse the data from the existing studies that focus on patients with heart failure, so as to better comprehend the clinical implications of the use of SGLT2 inhibitors. Additionally, we *a priori* planned to analyse the existing evidence depending on the diabetic status with the goal to determine the efficacy of SGLT2 both in the diabetic population and in heart failure patients without diabetes.

## 2. Methods

This is a systematic review and meta-analysis conducted and reported according to the Preferred Reporting Items for Systematic Reviews and Meta-Analysis (PRISMA) guidelines. It has been submitted and registered with PROSPERO (registration number: CRD42021224777).

### 2.1. Search Strategy

PubMed, Web of Science, and Cochrane library databases were systematically searched from inception until November 20^th^ 2020. The key terms used for the search were (“SGLT2” *or* “Sodium-glucose cotransporter-2 inhibitors” or “canagliflozin” or “dapagliflozin” or “empagliflozin” or “ertugliflozin”) and “heart failure.”

### 2.2. Study Selection

After removing duplicates, all the remaining studies were screened at the title/abstract level. Our inclusion criteria were as follows:

1. Observational or randomised controlled studies comparing SGLT2 inhibitors with placebo
2. Included adults (>18 years old)
3. Included patients diagnosed with heart failure, either with prespecified echocardiographic parameters (ejection fraction) or investigator-reported
4. Assessed mortality or clinical outcomes in patients with heart failure taking SGLT2 inhibitors
5. Studies which include imaging or blood biomarker parameters as outcomes that were included in the systematic review but not the meta-analysis

The selected studies underwent full-text screening. This process was performed by 3 independent investigators (R. C., M. D., and V. T.). Any conflicts were resolved by discussion, after which consensus was achieved. The study selection process is depicted in Figure 1 in Supplementary Materials.

### 2.3. Data Extraction

Two investigators (V. T. and R. B.) independently extracted the data from the selected studies using prespecified collection forms. The data extracted included type and characteristics of the study, number of patients on each group, number of diabetic and nondiabetic patients (where applicable), hazard ratios and confidence intervals for hospitalisation for heart failure (HHF), cardiovascular (CV) mortality, and all-cause mortality. The main outcomes of interest were all-cause mortality, CV mortality, and HHF. In studies that included both HF and non-HF patients, the data for the HF group with reduced ejection fraction was extracted from the prespecified subgroup analysis given in the respective study. Additionally, wherever possible, the outcomes were evaluated in subgroup analyses that included (1) patients with diabetes and (2) patients without diabetes.

### 2.4. Data Analysis

The hazard ratios and 95% CI that were given in each study were used for the meta-analysis. A random-effects model with inverse-variance weights was used to combine the effect measures from all studies on a logarithmic scale. Wherever possible, subgroup analysis in diabetic versus nondiabetic patients was performed. Statistical heterogeneity was assessed using the *I*^2^ statistic. The statistical analyses were conducted using the Review Manager (RevMan) software (version 5.3. Copenhagen: The Nordic Cochrane Centre, The Cochrane Collaboration, 2014). The statistical significance was defined as *p* < 0.05. The between-study variance component was estimated using the DerSimonian and Laird method, which is the default approach of the software used for this meta-analysis [9].

## 3. Results

Out of the 86 studies that underwent full-text evaluation, a total of 8 studies including 13,275 participants were included. A total of 6,877 of these participants were in the SGLT2 group, while 6,398 were in the placebo group. Dapagliflozin was used in three Randomised Control Trials (RCT), empagliflozin in two RCTs, and canagliflozin, sotagliflozin, and ertugliflozin were used in one RCT each. Some three studies included patients with and without diabetes, two of which provided hazard ratios for cardiovascular and mortality outcomes that were used in the statistical analyses [7, 8]. The nondiabetic cohort is comprised of 4,576 patients, out of which 2,284 were in the SGLT2 group and 2,292 were in the placebo group. While the majority of the studies had prespecified left ventricular ejection fraction (LVEF) in their inclusion criteria, two studies had “investigator reported HF” with no prerequisite for EF for inclusion in the study, while one required specific NT-proBNP level along with previous hospitalisation for HF. Three studies included patients with EF ≤ 40%, two studies included patients with EF ≤ 45% and EF > 45%, one study reports a median baseline EF of 35% and two studies did not have prespecified baseline ejection fraction of the heart failure population in their inclusion criteria. The two studies (DECLARE-TIMI 58 & VERTIS CV) that have included patients with both EF ≤ 45% and EF > 45% have provided separate hazard ratios for the two groups. In order to maintain a more homogeneous group of baseline characteristics, the hazard ratios given for the group with the EF < 45% were used in the meta-analyses of the efficacy endpoints for the total population examined in our review and in the subgroup analysis according to the baseline diabetes status. However, a further subgroup analysis was performed to examine the impact of SGLT2 inhibitors in patients with EF ≤ 45% compared with their impact in patients with EF > 45%.  Table 1 summarises the characteristics of all the studies included in the meta-analysis with the available number of cardiovascular events provided from each study, while Supplementary Table 1 summarises the cardiovascular outcomes for patients with and without diabetes from the studies that investigated these cohorts separately. Cochrane collaboration's tool was used for assessment of risk of bias to assess the randomised controlled studies (Supplementary Table 2).

### 3.1. Efficacy Endpoints

**Figure 1:**
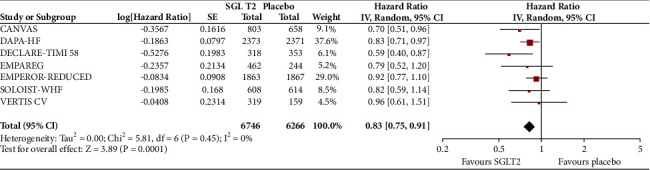
Effect of SGLT2 inhibitors versus placebo on all-cause mortality for the total population.

**Figure 2:**
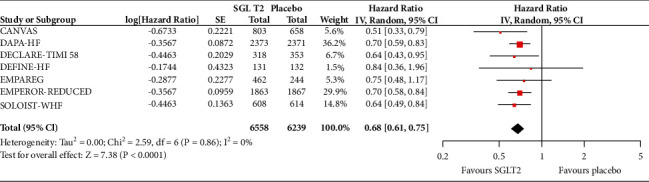
Effect of SGLT2 inhibitors versus placebo on hospitalisation for heart failure for the total population.

**Figure 3:**
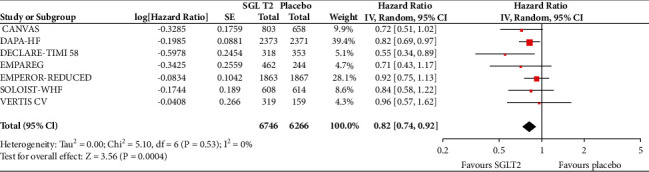
Effect of SGLT2 inhibitors versus placebo on cardiovascular death for the total population.

**Figure 4:**
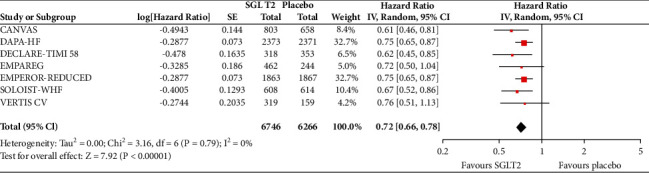
Effect of SGLT2 inhibitors versus placebo on hospitalisation for heart failure or cardiovascular death for the total population.

**Figure 5:**
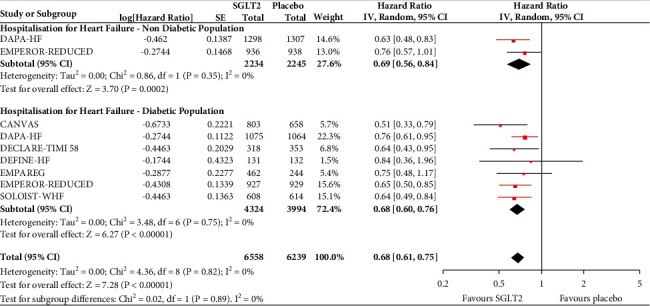
Subgroup analysis of the treatment effect SGLT2 inhibitors on hospitalisation for heart failure depending on baseline diabetes status.

**Figure 6:**
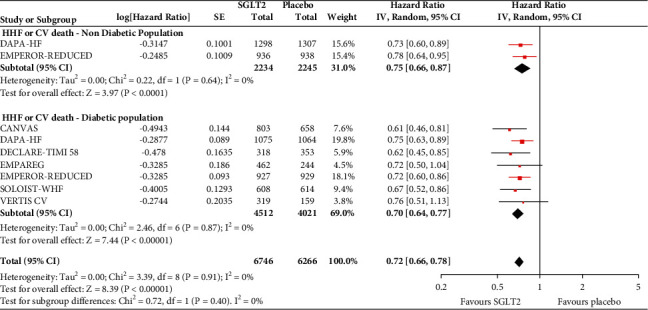
Subgroup analysis of the treatment effect SGLT2 inhibitors on hospitalisation for heart failure or cardiovascular death depending on baseline diabetes status.

**Figure 7:**
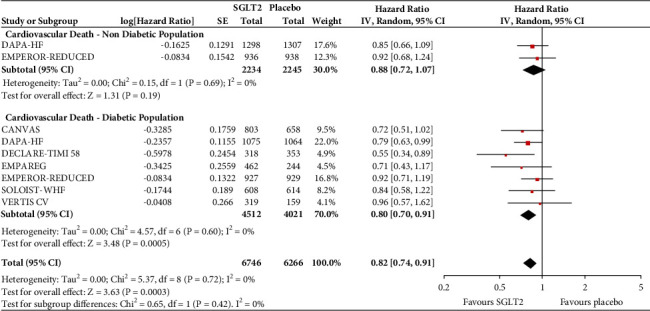
Subgroup analysis of the treatment effect SGLT2 inhibitors on cardiovascular death depending on baseline diabetes status.

In the analyses that included all the participants (regardless of diabetes status), the use of SGLT2 inhibitors was associated with significantly reduced risk of all-cause mortality (HR = 0.83, 95% CI, 0.75–0.91; *I*^2^ 0%) (Figure 1), hospitalisation for heart failure (HHF) (HR = 0.68, 95% CI, 0.61–0.75; *I*^2^ 0%) (Figure 2), CV death (HR = 0.82, 95% CI, 0.74–0.92; *I*^2^ 0%) (Figure 3), and hospitalisation for heart failure or CV death (HR = 0.72, 95% CI, 0.66–0.78; *I*^2^ 0%) (Figure 4) compared with placebo. Subgroup analysis of the total population according to the diabetes status showed that SGLT2 inhibitors significantly reduced the risk of HHF both in patients with and without diabetes (HR = 0.68, 95% CI: 0.60–0.76; *I*^2^ 0% and HR = 0.69, 95% CI: 0.56–0.84; *I*^2^ 0%, respectively) (Figure 5), as well as the risk of HHF or CV death (HR = 0.70, 95% CI: 0.64–0.77; *I*^2^ 0% and HR = 0.75, 95% CI: 0.66–0.87; *I*^2^ 0%, respectively) (Figure 6). The favourable impact of SGLT2 inhibitors on the outcome of CV death alone was significant in the participants with diabetes although it did not reach the level of statistical significance in the participants without diabetes (Figure 7). Additionally, subgroup analysis according to baseline EF was performed from the data available from the two studies (DECLARE-TIMI 58 & VERTIS CV) that stratified patients according to this. SGLT2 inhibitors did not have a significant impact on all-cause mortality (HR = 0.88, 95% CI: 0.67–1.14; *I*^2^ 45%) (Supplementary Figure 2) or cardiovascular death (HR = 0.95, 95% CI: 0.63–1.44; *I*^2^ 65%) (Supplementary Figure 3) with no significant differences between the two groups. However, SGLT2 inhibitors significantly reduced the risk of hospitalisation for heart failure or CV death (HR 0.78, 95% CI: 0.65–0.94; *I*^2^ 9%) with more pronounced effect on the group of heart failure with reduced ejection fraction, whereas in participants with EF > 45%, the favourable impact of SGLT2 inhibitors did not reach the level of statistical significance (Supplementary material Figure 4).

Funnel plots for unadjusted all-cause mortality (Supplementary Figure 5) and hospitalisation for heart failure (Supplementary Figure 6) were used to assess publication bias with no evidence of significant publication bias.

### 3.2. The Impact of SGLT2 on LV Function and BNP

A relatively small number of studies have been published so far investigating the impact of SGLT2 inhibitors on LV function and dimensions as assessed by imaging parameters, biomarkers (NT-proBNP or BNP), exercise capacity, symptom improvement, and quality of life in patients with heart failure.  Table 2 summarises these studies along with their main characteristics and outcomes of interest.

EMPA-TROPISM is the only study so far investigating the effect of SGLT2 exclusively in nondiabetic patients [10]. Comprised of 84 participants, it showed that empagliflozin had a significantly positive impact on LV function and remodelling as well as on the quality of life compared to placebo. On the other hand, the EMPIRE-HF study, which included 190 diabetic and nondiabetic patients, did not show significant differences between empagliflozin and placebo in any of the endpoints investigated (NT-proBNP, activity level and quality of life/symptomatic improvement) 3 months after initiation of the treatment [11]. While this RCT did not study LV function or volumes by any means of imaging, it is notable that the follow-up period was relatively short (3 months), which differentiates it from the rest of the studies. Apart from the relatively short follow-up period, the participants of this study were patients with a relatively milder phenotype of heart failure with better baseline status and functional capacity and lower baseline NT-proBNP levels [11].

LV function was assessed by echocardiography in some of the studies and by CMR in others. From the echocardiographic studies, it was noted that SGLT2 inhibitors did have a positive impact on diastolic function. In two of the three studies, the majority of the patients had HF with preserved ejection fraction [12, 13], while the third study included a small number of patients (twelve) with advanced/drug refractory heart failure [14]. Even in this small cohort with advanced disease, there was an improvement in the E/e' ratio; however, this did not reach the level of statistical significance (*p* value given as 0.06).

From the studies that utilised CMR, it may be argued that SGLT2 favours cardiac remodelling and improvement in LV volumes; however, results are not consistent. More specifically, the LV end-diastolic volume (LVEDV) was assessed with the use of CMR in three RCTs: SUGAR-DM-HF, EMPA-TROPISM, and REFORM [10, 15, 16]. SUGAR-DM-HF and EMPA-TROPISM included a combined number of 189 patients and demonstrated a significant improvement of the LVEDV in the SGLT2 arm compared to placebo [10, 15]. Remarkably, this positive effect was also noted in the nondiabetic cohort that the EMPA-TROPISM study included [10]. The REFORM study included 56 patients in total, and after 12 months of follow-up, the investigators did not find significant differences in the LV volumes between the SGLT2 and placebo groups [16]. It should be noted that the patient cohort consisted of diabetic patients with mild HF symptoms on modest doses of loop diuretics. Nevertheless, in the same study, a significant reduction in the diuretic requirements was noted in the SGLT2 group.

The evidence regarding BNP/NT-proBNP is somewhat inconsistent, with some of the studies showing improvement [13–15] and others demonstrating no substantial changes between the two groups [12, 17]. This could be explained by the discrepancies in the baseline characteristics of the participants in addition to the different follow-up periods and methodology in each study.

Despite the fact that each of the aforementioned studies comprised a relatively small number of patients and investigated LV function and parameters by different imaging modalities (echocardiography or CMR), it can be argued that even in this heterogeneous group, there is a general trend towards improvement of diastolic function and LV volumes in the SGLT2 group, even if these did not always reach the level of statistical significance. Further research with large randomised controlled trials would be beneficial in portraying the impact of SGLT2 inhibitors on LV function, tissue characterisation, and cardiac remodelling.

## 4. Discussion

The present meta-analysis, the largest to date, shows that the use of SGLT2 inhibitors is associated with reduction in the risk of hospitalisation for heart failure, cardiovascular death, and all-cause mortality in patients with heart failure primarily with reduced ejection fraction. In subgroup analyses stratified by the presence of diabetes, it is demonstrated that SGLT2 inhibitors provide consistent benefit on cardiovascular outcomes regardless of the baseline diabetes status.

Our data are in agreement with previous evidence that supports the prognostic value of SGLT2 in cardiovascular outcomes and mortality. Crucially, our findings highlight the importance of this drug group in the patients with heart failure regardless of diabetes status, revealing in this way that the potential therapeutic benefit in this population cohort could be invaluable.

### 4.1. Current Evidence and Recommendations

Previous large RCTs have shown remarkable benefits of SGLT2 specifically in cardiovascular outcomes in the diabetic population [18–20]. These results drew the attention to one or more potentially unrevealed thus far cardioprotective mechanisms of SGLT2 inhibitors that make it unique in the world of oral antidiabetic medications. Interestingly, the impact of SGLT2 inhibitors on cardiovascular outcomes does not seem to be directly related with their glucose-lowering efficacy [5]. Since there are now accumulating evidence supporting that the cardioprotective mechanisms are not associated with the glycemic control, the focus has been shifted to using these agents to patients regardless of their baseline diabetes status. Previous studies that have investigated the effect of SGLT2 inhibitors in nondiabetic cohorts have so far demonstrated positive results [7, 8, 10, 21]. The rapidly accumulating evidence of the substantial favourable impact of these agents on risk reduction in hospitalisation for heart failure and cardiovascular death has led in their inclusion in the latest recommendations for the management of patients with heart failure [22]. Notably, given the results of DAPA-HF and EMPEROR-Reduced trials, dapagliflozin and empagliflozin are now recommended in symptomatic patients with HF and reduced EF on optimal treatment, regardless of the presence of diabetes [7, 8, 22].

### 4.2. Potential Mechanisms of Action

While the exact pathophysiological process remains to be fully understood, there are several hypotheses that investigate the cardiometabolic profile of these agents. Some of the benefits observed most notably particularly in the HF population could be explained by the natriuresis and osmotic diuresis that these agents promote [5, 23]. This, subsequently, results in improvement of the left ventricular loading conditions by a reduction in the preload. While one may argue that this is a feature of all the commonly used diuretics, it has been noted that SGLT2 inhibitors do not reduce the intravascular volume as much as the common diuretics but instead target rather selectively the interstitial fluid, with a greater reduction in the extracellular fluid and no major impact on organ perfusion [24, 25]. Interestingly, in a small study by Griffin et al., it was demonstrated that empagliflozin resulted in natriuresis which was independent of the glucose load, indicating a direct natriuretic effect distinct from the osmotic diuresis [26]. Additionally, in contrast with the loop diuretics, SGLT2 inhibitors promote uricosuria and can reverse diuretic-induced hyperuricaemia, another contributing factor to their cardiovascular protective effects [27].

SGLT2 inhibitors have also been shown to reduce the blood pressure without increasing the heart rate and therefore improve the myocardial workload [28]. The pathophysiology behind this mechanism is not delineated yet; nevertheless, there is data to suggest that reduction in arterial stiffness and improvement on vascular resistance may play a significant role [5, 6, 28].

The action of SGLT2 inhibitors on a cellular level is surprising as they induce a state that mimics starvation [29]. As a result, there is activation of signaling pathways that involve important enzymes such as the sirtuin 1 (SIRT1) and the adenosine monophosphate-activated protein kinase (AMPK), both of which attenuate oxidative stress and inflammation and promote oxidation of fatty acids resulting in ketonaemia [29, 30]. Additionally, the activation of SIRT1 leads to stimulation of erythropoietin synthesis and erythrocytosis, which has been found to be one of the factors contributing to the significant cardiovascular benefits of SGLT2 inhibitors [31, 32].

Furthermore, SGLT2 inhibitors improve insulin sensitivity and glycemic control. With reduced requirements in insulin, SGLT2 inhibitors promote weight loss which also contributes to lower blood pressure [33]. While it could be argued that these effects are reflected on the improvement of diastolic function and filling pressure parameters noted in the echocardiographic studies investigating the impact of these agents on LV function, further research on this matter is required to prove this hypothesis.

Another promising emerging feature of SGLT2 inhibitors is their antifibrotic impact on the heart [34, 35]. Preclinical research data have demonstrated that empagliflozin directly attenuates cardiac myofibroblast activity and collagen remodelling [34], while dapagliflozin also diminishes the process of myocardial fibrosis after myocardial infarction [35]. It will be of great clinical interest to assess if these research data are in accordance with findings from CMR studies focusing on the left ventricular tissue characterisation.

Undoubtedly, CMR holds an important role in the assessment of LV function, and it can provide invaluable information about the impact of the SGLT2 inhibitors on left ventricular function and remodelling. Recently, the EMPA-HEART CardioLink-6 randomised placebo-controlled trial thoroughly investigated data from 74 patients with diabetes type 2 with coronary artery disease that underwent a comprehensive CMR study [36]. It demonstrated a significant reduction in myocardial extracellular compartment volume (ECV), indexed extracellular compartment volume (iECV), and indexed LV mass (LVMi) after 6 months of treatment with empagliflozin compared with placebo. In the same study, tissue remodelling biomarkers were also measured at baseline and at 6 months, with no significant difference found between the SGLT2 and placebo groups. It has to be noted however that this study was not powered to detect differences in these and that in this patient cohort the baseline levels of these biomarkers were actually in the normal range. Therefore, further studies, including ideally patients with heart failure, are required to obtain detailed information that could provide a new perspective in the mechanism of action of SGLT2 inhibitors on the diseased myocardium.

Independently of the mode of action, our meta-analysis conclusively confirms that SGLT2 inhibitors have a beneficial effect in patients with HF independently of the diabetes status, reducing mortality by 17%, and hospitalisation for heart failure by almost a third, supporting the need for increased utilisation in patients with reduced LVEF.

## 5. Limitations

This study has potential limitations that should be considered. Firstly, not all the randomised controlled trials have published the necessary subgroup data for all the endpoints. Therefore, some of these trials were not included in the analysis of individual endpoints. Secondly, in this meta-analysis we included RCTs that performed subgroup analysis depending on the HF status, regardless of their definition of HF. While most of the studies gave prespecified EF in their inclusion criteria, some studies had “investigator-reported HF.” Additionally, in order to maintain homogeneity, the data published for the cohort with the reduced EF were used in the meta-analyses of the efficacy endpoints for the total population, as this cohort represents the vast majority of the participants in the RCTs analysed in this meta-analysis. We performed subgroup analysis of the main endpoints according to the EF, when these data were provided. Whereas the level of heterogeneity for the analyses of the endpoints for the total population was insignificant as assessed by *I*^2^ of 0%, there was moderate to substantial heterogeneity in the subgroup analyses of endpoints according to EF as evidenced by an *I*^2^ that ranged between 9% and 65%. It has to be acknowledged that the subgroup analysis of endpoints according to baseline EF was comprised of only two studies and a small number of individuals, and this could be one of the reasons for the higher level of heterogeneity. Finally, we acknowledge that assessment of publication bias with the use of funnel plots is less reliable when the meta-analysis is comprised of less than ten studies in total.

## 6. Conclusion

It is without a doubt that SGLT2 inhibitors provide prognostic benefit in patients with heart failure, regardless of the exact mechanism of action. The recent large RCTs have shown that this positive impact is expanded in patients without diabetes. This systematic review and meta-analysis provide robust summative evidence of the effectiveness of these agents in patients with heart failure regardless of the diabetes status. Our results also suggest that they are likely to be more effective in patients with reduced LVEF. Data on their mechanism is limited with imaging studies performed to date providing conflicting information. Further studies are needed to better understand their mechanisms of action and their long-term impact on LV function and biomarkers as well as the heart failure phenotype that will benefit most from them. Nevertheless, given the unique pathophysiological profile of SGLT2 inhibitors and their significant benefit in cardiovascular profile, they have an invaluable role in the management of patients with heart failure. The role of CMR is critical in facilitating volumes and tissue characterisation, and it will take a prominent role in future research studies.

## Figures and Tables

**Table 1:** Characteristics of studies included in the meta-analysis.

Trial	SGLT2	Definition of HF at baseline	Diabetes status of participants	Number of participants		HHF events		CV death		All-cause mortality		CV death or HF	
				SGLT2	Placebo	SGLT2	Placebo	SGLT2	Placebo	SGLT2	Placebo	SGLT2	Placebo
Emperor-reduced	Empagliflozin	EF ≤ 40%	DM + non-DM	1863	1867	388	553	187	202	249	266	361	462
Soloist-WHF	Sotagliflozin	Previous hospitalisation for HF and BNP > 150pg/ml (>450pg/ml for AF) (reported median EF 35%)	DM	608	614	194	297	51	58	65	76	245	355
Vertis-CV‡	Ertugliflozin	EF ≤ 45%‡	DM	319	159	—	—	42	21	54	27	62	38
		EF > 45%		680	327			47	21	63	30	68	35
Empareg	Empagliflozin	Investigator reported HF	DM	462	244	48	30	38	27	56	35	75	49
Declare-Timi 58 (HF)‽	Dapagliflozin	EF ≤ 45%	DM	318	353	41	63	25	47	38	68	59	95
		EF > 45%						54	38	84	81	92	99
Dapa-HF	Dapagliflozin	EF ≤ 40%	DM + non-DM	2373	2371	231	318	227	273	276	329	382	495
Define-HF	Dapagliflozin	EF ≤ 40%	DM + non-DM	131	132	12	13	—	—	—	—	—	—
CanvasS	Canagliflozin	Investigator reported HF	DM	803	658	—	—	—	—	—	—	—	—

^‡^Only the group with EF <45% and known history of HF was analysed in the total population analysis and in the subgroup analysis according to baseline diabetes status. ^?^The subgroup of HF with reduced ejection fraction was analysed in the total population analysis and in the subgroup analysis according to baseline diabetes status.

**Table 2:** Review of studies on SGLT2 and LV function.

Study (authors and name of trial where applicable)	Study design	Number of participants	Baseline HF status	Diabetes status of participants	Follow-up period	Study endpoints	Outcomes
Lee et al. Sugar-DM-HF	RCT (empagliflozin versus placebo)	105	EF ≤ 40%	DM prediabetes	9 months	Primary: difference in change of LVESVi & GLS Secondary: difference in change of LVEF, LVEDVi, NT-proBNP, 6MWT, KCCQ-TSS (all imaging parameters assessed by CMR)	Significant improvement in LVESVi, LVEDVi, NT-proBNP in the empagliflozin group compared to placebo. No difference in GLS, LVEF, 6MWT, and KCCQ-TSS between the groups.

Jensen et al. Empire-HF	RCT (empagliflozin versus placebo)	190	EF ≤ 40%	DM Non-DM	3 months	Primary: difference in change of NT-proBNP Secondary: daily activity level, KCCQ-OSS	No differences noted between the groups in the change of NT-proBNP, daily activity level or KCCQ-OSS

Santos-Gallego et al. Empa-tropism	RCT (empagliflozin versus placebo)	84	EF < 50%	Non-DM	6 months	Primary: difference in change of LVEDV and LVESV Secondary: difference in change in peak VO2 (assessed by CPET), LVM, LVEF, 6MWT, and KCCQ-12 (all imaging parameters assessed by CMR)	Significant improvement of all the study endpoints (primary and secondary) in the empagliflozin group

Singh et al. REFORM	RCT (dapagliflozin versus placebo)	56	EF < 45%	DM	12 months	Primary: difference in change of LVESV Secondary: LVEDV, LVMi, and LVEF (all imaging parameters assessed by CMR)	No differences between the groups in the change of LVESV, LVEDV, LVMi, and LVEF

Tanaka et al.	Prospective multicentre study (dapagliflozin)	53	HFpEF and HFrEF (majority HFpEF)	DM	6 months	Primary: diastolic function (E/e'), GLS Secondary: LVEDV, LVESV, LVEF, LVMi, LAVi, and BNP (all imaging parameters assessed by 2D echocardiography)	Dapagliflozin was associated with improvement in diastolic function (E/e') and GLS as well as LAVi. No significant changes in the rest of the parameters studied in the 6-month follow-up period

Seo et al.	Retrospective study (empagliflozin, canagliflozin, dapagliflozin)	12	Advanced/drug-refractory HF	DM	6 months	NYHA class, BNP, LVEDV, LVEF, E/e', TRPG (all imaging parameters assessed by 2D echocardiography)	Improvement was noted in NYHA class, LVEDV, TRPG, and BNP levels 6 months after initiation of the SGLT2. No changes in the rest of the parameters studied in the 6-month follow-up period

Sezai et al. Canossa	Prospective controlled trial (canagliflozin)	35	HFpEF and HFrEF (majority HFpEF)	DM	12 months	Primary: changes of subcutaneous, visceral, and total fat areas (determined by computed tomography) Secondary: ANP, BNP, LVEF, LVMi, diastolic function (E/e') (amongst others) (all imaging parameters assessed by 2D echocardiography)	All fat areas significantly decreased after 12 months treatment with SGLT2. ANP, BNP, LVEF, LVMi, and E/e' also significantly improved

RCT, randomised controlled trial; HFpEF, heart failure with preserved ejection fraction; HFrEF, heart failure with reduced ejection fraction; EF, ejection fraction; DM, diabetes mellitus; LVESVi, left ventricular end systolic volume indexed; LVEDVi, left ventricular end-diastolic volume indexed; GLS, global longitudinal strain; LVEF, left ventricular ejection fraction; LVMI, left ventricular mass indexed; LAVi, left atrial volume indexed; 6MWT, 6-minute walk test; KCCQ-TSS, Kansas City Cardiomyopathy Questionnaire Total Symptom Score; KCCQ-OSS, Kansas City Cardiomyopathy Questionnaire Overall Summary Score; CPET, cardiopulmonary exercise test; E/E', ratio of early diastolic peak velocity of Doppler transmitral flow to early diastolic mitral annular velocity; TRPG, pressure gradient of tricuspid regurgitation; ANP, atrial natriuretic peptide; BNP, brain natriuretic peptide.

## Data Availability

Previously reported data were used to support this meta-analysis. These prior studies (and datasets) are cited at relevant places within the text as references.

## Supplementary Materials

Supplementary Materials *Supplementary Table 1.* Characteristics of the studies included in the meta-analysis that have outcomes for the patients with and without diabetes. *Supplementary Table 2.* Cochrane Collaboration's tool for assessing risk of bias in randomised controlled trials. *Supplementary Figure 1.* PRISMA flow diagram of the study selection progress. *Supplementary Figure 2.* Subgroup analysis of the treatment effect of SGLT2 inhibitors on all-cause mortality depending on baseline ejection fraction. *Supplementary Figure 3.* Subgroup analysis of the treatment effect SGLT2 inhibitors on cardiovascular death depending on baseline ejection fraction. *Supplementary Figure 4.* Subgroup analysis of the treatment effect SGLT2 inhibitors on risk of hospitalisation for heart failure (HHF) or cardiovascular death depending on baseline ejection fraction. *Supplementary Figure 5.* Funnel plot for unadjusted all-cause mortality demonstrating no evidence of significant publication bias. *Supplementary Figure 6.* Funnel plot for unadjusted risk of hospitalisation for heart failure demonstrating no evidence of significant publication bias.
