# Supplementary material for: The Role of SGLT2 Inhibitors in Heart Failure: A Systematic Review and Meta-Analysis
Source: Cardiol Res Pract. 2021 Aug 19;2021:9927533. doi: 10.1155/2021/9927533 (PMC8397556; doi:10.1155/2021/9927533)

Supplementary Figure 2. Subgroup analysis of the treatment effect SGLT2 inhibitors on all-cause mortality depending on baseline ejection fraction

Study or Subgroup	log[Hazard Ratio]	SE	SGLT2 Total	Placebo Total	Weight	Hazard Ratio IV, Random, 95% CI
**All cause mortality - HFpEF**						
DECLARE-TIMI 58	0.0198	0.1569	84	81	31.5%	1.02 [0.75, 1.39]
VERTIS CV	0.01	0.2171	63	30	22.6%	1.01 [0.66, 1.55]
**Subtotal (95% CI)**			**147**	**111**	**54.1%**	**1.02 [0.79, 1.30]**
Heterogeneity: Tau² = 0.00; Chi² = 0.00, df = 1 (P = 0.97); I² = 0%						
Test for overall effect: Z = 0.13 (P = 0.90)						
**All cause mortality - HFrEF**						
DECLARE-TIMI 58	-0.5276	0.1983	38	68	25.0%	0.59 [0.40, 0.87]
VERTIS CV	-0.0408	0.2314	54	27	20.9%	0.96 [0.61, 1.51]
**Subtotal (95% CI)**			**92**	**95**	**45.9%**	**0.74 [0.46, 1.19]**
Heterogeneity: Tau² = 0.07; Chi² = 2.55, df = 1 (P = 0.11); I² = 61%						
Test for overall effect: Z = 1.23 (P = 0.22)						
**Total (95% CI)**			**239**	**206**	**100.0%**	**0.88 [0.67, 1.14]**
Heterogeneity: Tau² = 0.03; Chi² = 5.49, df = 3 (P = 0.14); I² = 45%						
Test for overall effect: Z = 0.99 (P = 0.32)						
Test for subgroup differences: Chi² = 1.32, df = 1 (P = 0.25), I² = 24.3%						

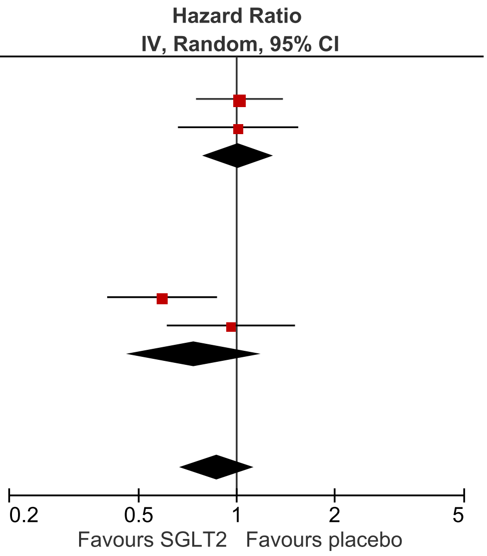

Supplement: Supplementary Materials — Supplementary Table 1. Characteristics of the studies included in the meta-analysis that have outcomes for the patients with and without diabetes. Supplementary Table 2. Cochrane Collaboration's tool for assessing risk of bias in randomised controlled trials. Supplementary Figure 1. PRISMA flow diagram of the study selection progress. Supplementary Figure 2. Subgroup analysis of the treatment effect of SGLT2 inhibitors on all-cause mortality depending on baseline ejection fraction. Supplementary Figure 3. Subgroup analysis of the treatment effect SGLT2 inhibitors on cardiovascular death depending on baseline ejection fraction. Supplementary Figure 4. Subgroup analysis of the treatment effect SGLT2 inhibitors on risk of hospitalisation for heart failure (HHF) or cardiovascular death depending on baseline ejection fraction. Supplementary Figure 5. Funnel plot for unadjusted all-cause mortality demonstrating no evidence of significant publication bias. Supplementary Figure 6. Funnel plot for unadjusted risk of hospitalisation for heart failure demonstrating no evidence of significant publication bias. [file 9927533.f1.zip › 9927533.f1/Supplementary Figure 2.pdf]
