# Supplementary material for: The Role of SGLT2 Inhibitors in Heart Failure: A Systematic Review and Meta-Analysis
Source: Cardiol Res Pract. 2021 Aug 19;2021:9927533. doi: 10.1155/2021/9927533 (PMC8397556; doi:10.1155/2021/9927533)

Supplementary Information

Supplementary Table 1.Characteristics of the studies included in the meta-analysis that have outcomes for the patients with and without diabetes

Supplementary Table 2. Cochrane Collaboration's tool for assessing risk of bias in randomized controlled trials

Supplementary Figure 1. PRISMA flow diagram of the study selection process

Supplementary Figure 2. Subgroup analysis of the treatment effect SGLT2 inhibitors on all-cause mortality depending on baseline ejection fraction

Supplementary Figure 3. Subgroup analysis of the treatment effect SGLT2 inhibitors on cardiovascular death depending on baseline ejection fraction

Supplementary Figure 4. Subgroup analysis of the treatment effect SGLT2 inhibitors on risk of hospitalisation for heart failure (HHF) or cardiovascular death depending on baseline ejection fraction

Supplementary Figure 5. Funnel plot for unadjusted all-cause mortality demonstrating no evidence of significant publication bias

Supplementary Figure 6. Funnel plot for unadjusted risk of hospitalisation for heart failure demonstrating no evidence of significant publication bias

Supplementary Table 1 Characteristics of the studies included in the meta-analysis that have outcomes for the patients with and without diabetes

**Trial**	**SGLT2**	**Number of participants**		**HHF events**		**CV death**		**All-cause mortality**		**CV death or HHF**	
		SGLT2	Placebo	SGLT2	Placebo	SGLT2	Placebo	SGLT2	Placebo	SGLT2	Placebo
**EMPEROR-REDUCED**	Diabetes	927	929	221	337	104	113	-	-	200	265
	No diabetes	936	938	167	216	83	89	-	-	161	197
**DAPA – HF**	Diabetes	1075	1064	138	172	121	148	143	178	213	268
	No diabetes	1298	1307	93	146	106	125	133	151	169	227

Supplementary Table 2 Cochrane Collaboration's tool for assessing risk of bias in randomised controlled trials

Trial	Sequence generation	Allocation concealment	Performance bias	Detection bias	Attrition bias	Reporting bias
**EMPEROR-REDUCED**	Low	Low	Low	Low	Low	Low
**SOLOIST-WHF**	Low	Low	Low	Low	Low	Low
**VERTIS-CV**	Low	Low	Low	Low	Low	Low
**EMPAREG**	Low	Low	Low	Low	Low	Low
**DECLARE-TIMI 58 (HF)**	Low	Low	Low	Low	Low	Low
**DAPA – HF**	Low	Low	Low	Low	Low	Low
**DEFINE – HF**	Low	Low	Low	Low	Medium	Low
**CANVAS**	Low	Low	Low	Low	Low	Low

Supplementary Figure 1. PRISMA flow diagram of the study selection process

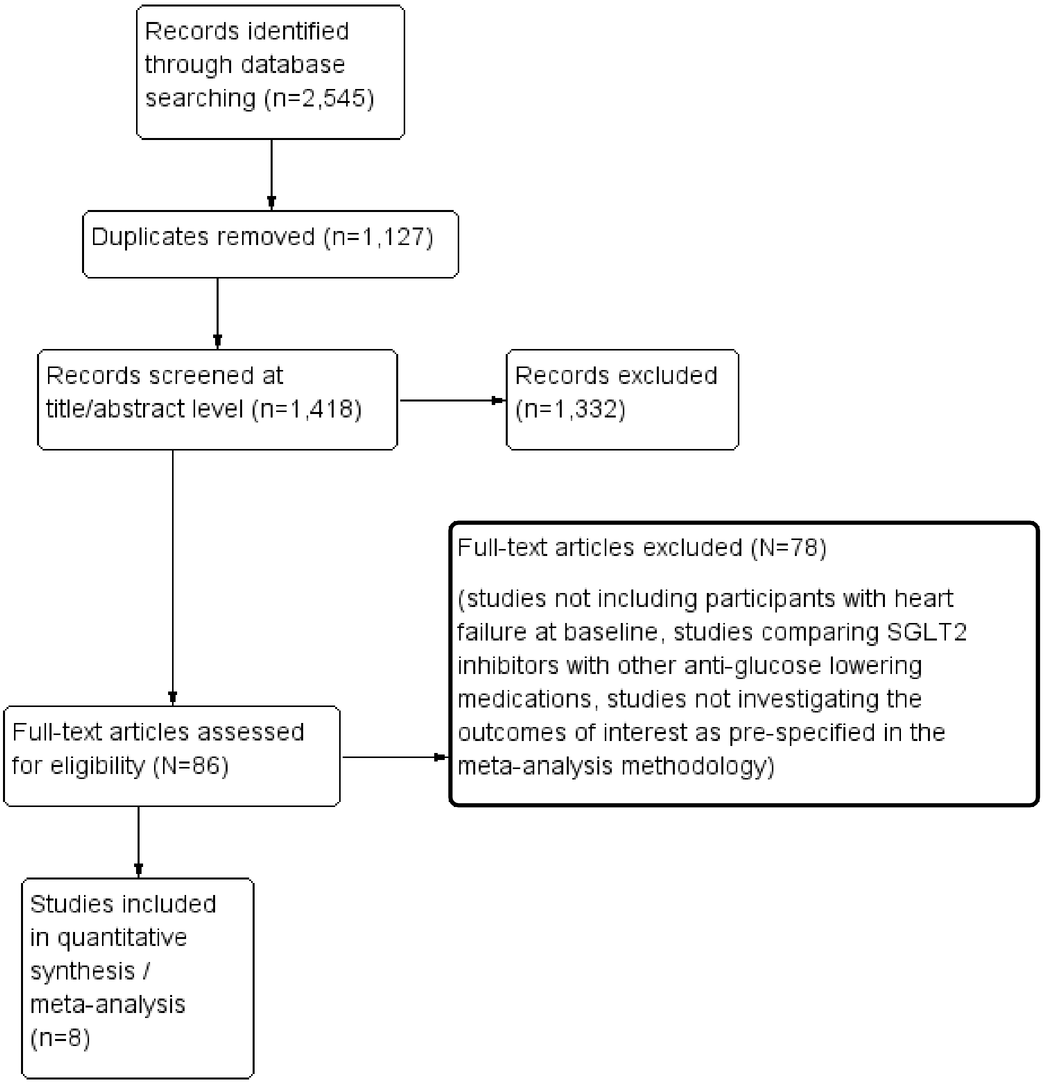

Supplementary Figure 2. Subgroup analysis of the treatment effect SGLT2 inhibitors on all-cause mortality depending on baseline ejection fraction

Study or Subgroup	log[Hazard Ratio]	SE	SGLT2 Total	Placebo Total	Weight	Hazard Ratio IV, Random, 95% CI
**All cause mortality - HFpEF**						
DECLARE-TIMI 58	0.0198	0.1569	84	81	31.5%	1.02 [0.75, 1.39]
VERTIS CV	0.01	0.2171	63	30	22.6%	1.01 [0.66, 1.55]
**Subtotal (95% CI)**			**147**	**111**	**54.1%**	**1.02 [0.79, 1.30]**
Heterogeneity: Tau² = 0.00; Chi² = 0.00, df = 1 (P = 0.97); I² = 0%						
Test for overall effect: Z = 0.13 (P = 0.90)						
**All cause mortality - HFrEF**						
DECLARE-TIMI 58	-0.5276	0.1983	38	68	25.0%	0.59 [0.40, 0.87]
VERTIS CV	-0.0408	0.2314	54	27	20.9%	0.96 [0.61, 1.51]
**Subtotal (95% CI)**			**92**	**95**	**45.9%**	**0.74 [0.46, 1.19]**
Heterogeneity: Tau² = 0.07; Chi² = 2.55, df = 1 (P = 0.11); I² = 61%						
Test for overall effect: Z = 1.23 (P = 0.22)						
**Total (95% CI)**			**239**	**206**	**100.0%**	**0.88 [0.67, 1.14]**
Heterogeneity: Tau² = 0.03; Chi² = 5.49, df = 3 (P = 0.14); I² = 45%						
Test for overall effect: Z = 0.99 (P = 0.32)						
Test for subgroup differences: Chi² = 1.32, df = 1 (P = 0.25), I² = 24.3%						

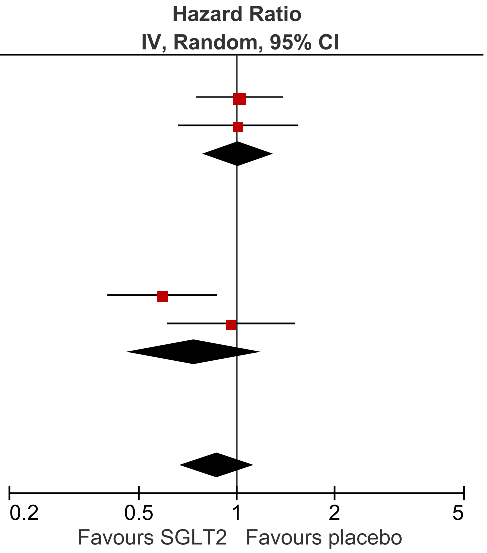

Supplementary Figure 3. Subgroup analysis of the treatment effect SGLT2 inhibitors on cardiovascular death depending on baseline ejection fraction

Study or Subgroup	log[Hazard Ratio]	SE	SGLT2 Total	Placebo Total	Weight	Hazard Ratio IV, Random, 95% CI
**Cardiovascular death - HFpEF**						
DECLARE-TIMI 58	0.3436	0.2123	54	38	27.5%	1.41 [0.93, 2.14]
VERTIS CV	0.077	0.267	47	21	23.6%	1.08 [0.64, 1.82]
**Subtotal (95% CI)**			**101**	**59**	**51.2%**	**1.27 [0.92, 1.76]**
Heterogeneity: Tau² = 0.00; Chi² = 0.61, df = 1 (P = 0.43); I² = 0%						
Test for overall effect: Z = 1.45 (P = 0.15)						
**Cardiovascular death - HFrEF**						
DECLARE-TIMI 58	-0.5978	0.2454	25	47	25.1%	0.55 [0.34, 0.89]
VERTIS CV	-0.0408	0.266	42	21	23.7%	0.96 [0.57, 1.62]
**Subtotal (95% CI)**			**67**	**68**	**48.8%**	**0.72 [0.42, 1.24]**
Heterogeneity: Tau² = 0.09; Chi² = 2.37, df = 1 (P = 0.12); I² = 58%						
Test for overall effect: Z = 1.18 (P = 0.24)						
**Total (95% CI)**			**168**	**127**	**100.0%**	**0.95 [0.63, 1.44]**
Heterogeneity: Tau² = 0.11; Chi² = 8.61, df = 3 (P = 0.03); I² = 65%						
Test for overall effect: Z = 0.23 (P = 0.82)						
Test for subgroup differences: Chi² = 3.08, df = 1 (P = 0.08), I² = 67.6%						

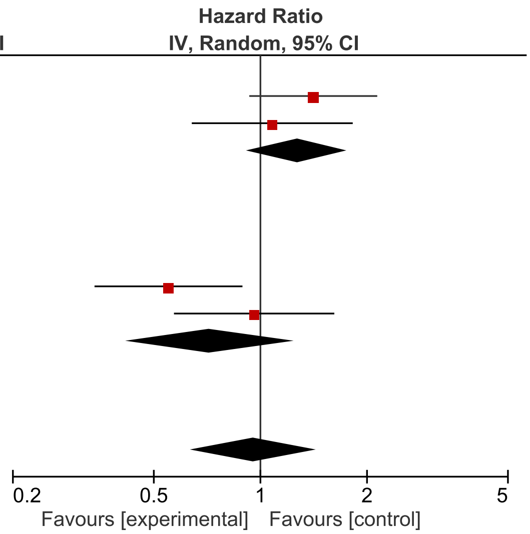

Supplementary Figure 4. Subgroup analysis of the treatment effect SGLT2 inhibitors on risk of hospitalisation for heart failure (HHF) or cardiovascular death depending on baseline ejection fraction

Study or Subgroup	log[Hazard Ratio]	SE	SGLT2 Total	Placebo Total	Weight	Hazard Ratio IV, Random, 95% CI
**HHF or Cardiovascular death - HFpEF**						
DECLARE-TIMI 58	-0.1278	0.1468	92	99	34.5%	0.88 [0.66, 1.17]
VERTIS CV	-0.0834	0.2097	68	35	18.0%	0.92 [0.61, 1.39]
**Subtotal (95% CI)**			**160**	**134**	**52.5%**	**0.89 [0.71, 1.13]**
Heterogeneity: Tau² = 0.00; Chi² = 0.03, df = 1 (P = 0.86); I² = 0%						
Test for overall effect: Z = 0.94 (P = 0.35)						
**HHF or Cardiovascular death - HFrEF**						
DECLARE-TIMI 58	-0.478	0.1635	59	95	28.5%	0.62 [0.45, 0.85]
VERTIS CV	-0.2744	0.2035	62	38	19.0%	0.76 [0.51, 1.13]
**Subtotal (95% CI)**			**121**	**133**	**47.5%**	**0.67 [0.52, 0.86]**
Heterogeneity: Tau² = 0.00; Chi² = 0.61, df = 1 (P = 0.44); I² = 0%						
Test for overall effect: Z = 3.12 (P = 0.002)						
**Total (95% CI)**			**281**	**267**	**100.0%**	**0.78 [0.65, 0.94]**
Heterogeneity: Tau² = 0.00; Chi² = 3.28, df = 3 (P = 0.35); I² = 9%						
Test for overall effect: Z = 2.69 (P = 0.007)						
Test for subgroup differences: Chi² = 2.64, df = 1 (P = 0.10), I² = 62.2%						

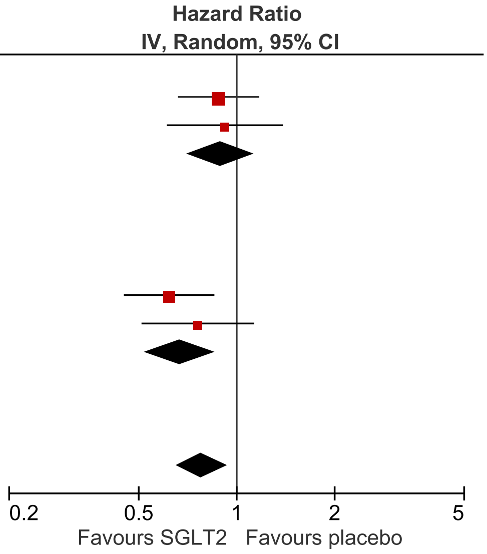

Supplementary Figure 5. Funnel plot for unadjusted all-cause mortality demonstrating no evidence of significant publication bias

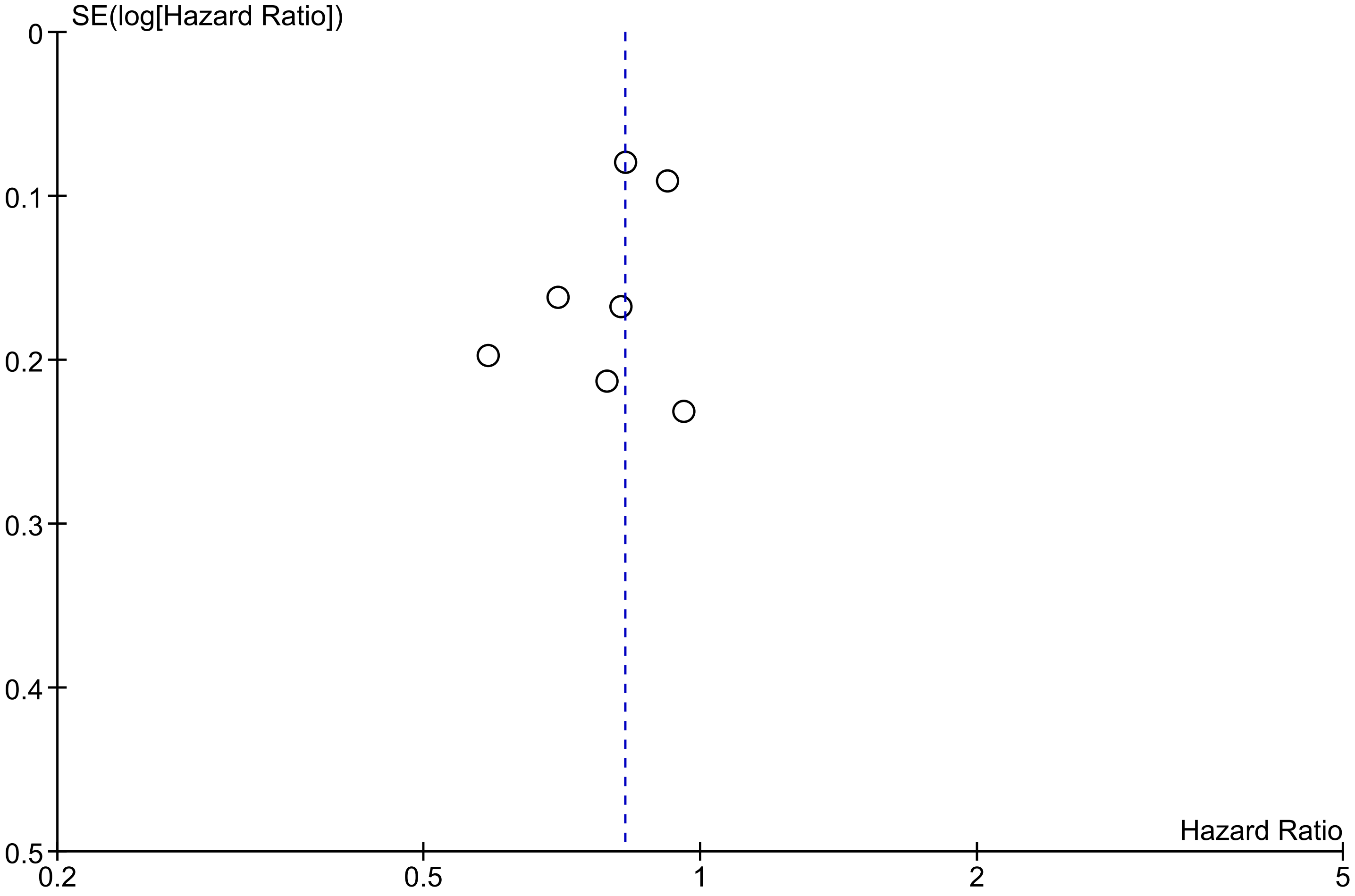

Supplementary Figure 6. Funnel plot for unadjusted risk of hospitalisation for heart failure demonstrating no evidence of significant publication bias

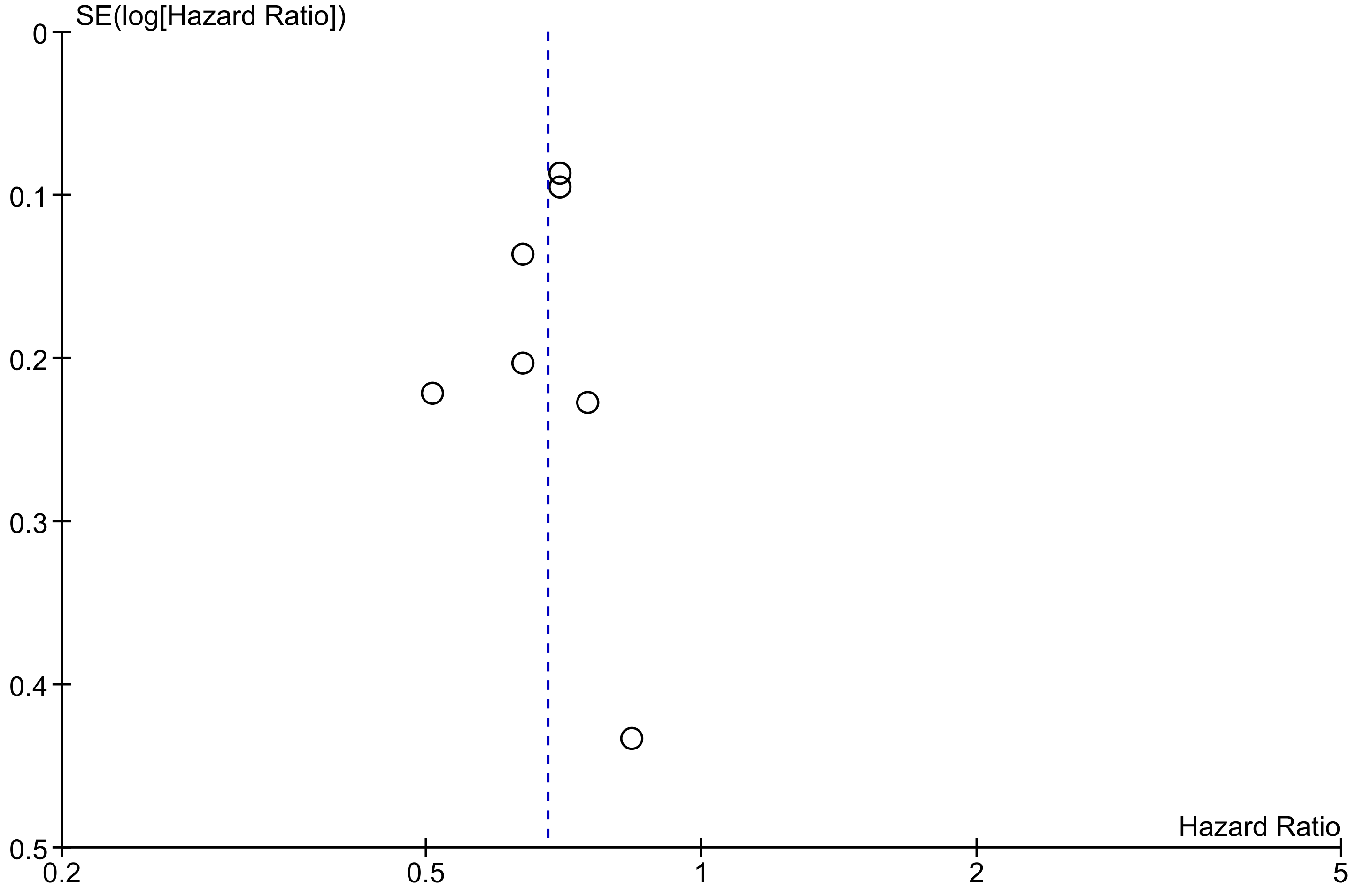

Supplement: Supplementary Materials — Supplementary Table 1. Characteristics of the studies included in the meta-analysis that have outcomes for the patients with and without diabetes. Supplementary Table 2. Cochrane Collaboration's tool for assessing risk of bias in randomised controlled trials. Supplementary Figure 1. PRISMA flow diagram of the study selection progress. Supplementary Figure 2. Subgroup analysis of the treatment effect of SGLT2 inhibitors on all-cause mortality depending on baseline ejection fraction. Supplementary Figure 3. Subgroup analysis of the treatment effect SGLT2 inhibitors on cardiovascular death depending on baseline ejection fraction. Supplementary Figure 4. Subgroup analysis of the treatment effect SGLT2 inhibitors on risk of hospitalisation for heart failure (HHF) or cardiovascular death depending on baseline ejection fraction. Supplementary Figure 5. Funnel plot for unadjusted all-cause mortality demonstrating no evidence of significant publication bias. Supplementary Figure 6. Funnel plot for unadjusted risk of hospitalisation for heart failure demonstrating no evidence of significant publication bias. [file 9927533.f1.zip › 9927533.f1/Supplementary Information.pdf]
