# Supplementary material for: The Role of SGLT2 Inhibitors in Heart Failure: A Systematic Review and Meta-Analysis
Source: Cardiol Res Pract. 2021 Aug 19;2021:9927533. doi: 10.1155/2021/9927533 (PMC8397556; doi:10.1155/2021/9927533)
Supplement: Supplementary Materials — Supplementary Table 1. Characteristics of the studies included in the meta-analysis that have outcomes for the patients with and without diabetes. Supplementary Table 2. Cochrane Collaboration's tool for assessing risk of bias in randomised controlled trials. Supplementary Figure 1. PRISMA flow diagram of the study selection progress. Supplementary Figure 2. Subgroup analysis of the treatment effect of SGLT2 inhibitors on all-cause mortality depending on baseline ejection fraction. Supplementary Figure 3. Subgroup analysis of the treatment effect SGLT2 inhibitors on cardiovascular death depending on baseline ejection fraction. Supplementary Figure 4. Subgroup analysis of the treatment effect SGLT2 inhibitors on risk of hospitalisation for heart failure (HHF) or cardiovascular death depending on baseline ejection fraction. Supplementary Figure 5. Funnel plot for unadjusted all-cause mortality demonstrating no evidence of significant publication bias. Supplementary Figure 6. Funnel plot for unadjusted risk of hospitalisation for heart failure demonstrating no evidence of significant publication bias. [file 9927533.f1.zip › 9927533.f1/Supplementary Table 1.pdf]

Supplementary Table 1 Characteristics of the studies included in the meta-analysis that have outcomes for the patients with and without diabetes

**Trial**	**SGLT2**	**Number of participants**		**HHF events**		**CV death**		**All-cause mortality**		**CV death or HHF**	
		SGLT2	Placebo	SGLT2	Placebo	SGLT2	Placebo	SGLT2	Placebo	SGLT2	Placebo
**EMPEROR-REDUCED**	Diabetes	927	929	221	337	104	113	-	-	200	265
	No diabetes	936	938	167	216	83	89	-	-	161	197
**DAPA – HF**	Diabetes	1075	1064	138	172	121	148	143	178	213	268
	No diabetes	1298	1307	93	146	106	125	133	151	169	227
